# Temporal profile of body temperature in acute ischemic stroke: relation to stroke severity and outcome

**DOI:** 10.1186/1471-2377-12-123

**Published:** 2012-10-18

**Authors:** Bartosz Karaszewski, Ralph GR Thomas, Martin S Dennis, Joanna M Wardlaw

**Affiliations:** 1 Division of Clinical Neurosciences, University of Edinburgh, Western General Hospital, Crewe Rd, Edinburgh, EH4 2XU, UK; 2 Department of Adult Neurology, Medical University of Gdans, , Poland; 3 SINAPSE Collaboration, Brain Research Imaging Centre, Division of Clinical Neurosciences, Western General Hospital, Crewe Rd, Edinburgh, EH4 2XU, UK

**Keywords:** Ischemic stroke, Tympanic body temperature, Pyrexia, Outcome, OCSP

## Abstract

**Background:**

Pyrexia after stroke (temperature ≥37.5°C) is associated with poor prognosis, but information on timing of body temperature changes and relationship to stroke severity and subtypes varies.

**Methods:**

We recruited patients with acute ischemic stroke, measured stroke severity, stroke subtype and recorded four-hourly tympanic (body) temperature readings from admission to 120 hours after stroke. We sought causes of pyrexia and measured functional outcome at 90 days. We systematically summarised all relevant previous studies.

**Results:**

Amongst 44 patients (21 males, mean age 72 years SD 11) with median National Institute of Health Stroke Score (NIHSS) 7 (range 0–28), 14 had total anterior circulation strokes (TACS). On admission all patients, both TACS and non-TACS, were normothermic (median 36.3°C vs 36.5°C, p=0.382 respectively) at median 4 hours (interquartile range, IQR, 2–8) after stroke; admission temperature and NIHSS were not associated (r^2^=0.0, p=0.353). Peak temperature, occurring at 35.5 (IQR 19.0 to 53.8) hours after stroke, was higher in TACS (37.7°C) than non-TACS (37.1°C, p<0.001) and was associated with admission NIHSS (r^2^=0.20, p=0.002). Poor outcome (modified Rankin Scale ≥3) at 90 days was associated with higher admission (36.6°C vs. 36.2°C p=0.031) and peak (37.4°C vs. 37.0°C, p=0.016) temperatures. Sixteen (36%) patients became pyrexial, in seven (44%) of whom we found no cause other than the stroke.

**Conclusions:**

Normothermia is usual within the first 4 hours of stroke. Peak temperature occurs at 1.5 to 2 days after stroke, and is related to stroke severity/subtype and more closely associated with poor outcome than admission temperature. Temperature-outcome associations after stroke are complex, but normothermia on admission should not preclude randomisation of patients into trials of therapeutic hypothermia.

## Background

Elevated body temperature (pyrexia) is said to be common after stroke [1,2] and is associated with poor outcome [2-10]. However, the timing of pyrexia after stroke varied between studies (Table 1). Pyrexia occurring early might reflect the systemic response to ischemic brain damage [11] whereas pyrexia occurring later might be due to complications of severe stroke, such as infection and deep venous thrombosis (DVT). However data on timing, causes and associations between body temperature and outcome vary.

**Table 1:** Previous studies of body temperature and outcome after stroke - methods

**First author and year**	**Study design**	**N**	**Types of stroke**	**Temperature measurement method**	**Time of 1**^**st**^**; interval; last reading**	**Statistical analysis**	**Outcome measures**	**Definition of pyrexia**
**Azzimondi 1995**[2]	P	183	Any stroke <48 hrs (not SAH)	A	N/S; 12 h; 7 d	Max temp in 7 d, logistic regression	CNS, serial GCS up to 30 d, 1, 3 & 6 month Barthel	≤37.2 = absence of fever. ≥37.9 = high fever
**Reith 1996**[3]	P	390	Any stroke <6 hrs	T	<6 h; -; -	Logistic regression	Lesion size, SSS, presence of infection, WCC	>37.5
**Castillo 1998**[9]	P	297	Ischaemic	A	<24 h; 2 h; 72 h	Correlate peak temp with clinical outcome and final infarct vol. Stepwise logistic regression	CSS, presence of infection, 4–7 d lesion volume, 3 month Barthel	>37.5
**Georgilis 1999**[4]	R	330	Any stroke	Most A, some RC	<48 h; 3 h; -	Presence/absence of fever and infection. Stepwise logistic regression	GCS, SSS, CT lesion volume, presence of infection, use of invasive procedures	>37.5 on >2 occasions on 2 consec. days
**Wang 2000**[12]	R	437	Any “acute” stroke	T	“Admission” but no time limit given; -; -	Logistic regression. ischaemic vs. haemorrhagic stroke	Co-morbidities, WCC, [glucose], mortality (in-hospital and 1 yr)	>37.5
**Boysen 2001**[8]	P	584	Ischaemic	T	<6 h; 2–4 h; 48 h	Mean temp analysis by subgroup	SSS, 3 month mRS	>37.5
**Kammersgaard 2002**[6]	P	390	Any stroke	T	<6 h; -; -	Dichotomised normothermia vs. pyrexia, and multivariate survival analysis	SSS, [glucose], 5 yr mortality	>37
**Audebert 2004**[13]	R	346	Ischaemic; Excl pts with infection pre- or post- stroke	O or RC	<24 h (mean 6.7 h); 2–12 h; 3 d	Subgroup analysis of median temp days 1-3	NIHSS, WCC, CRP, 1–5 d lesion volume (CT/MR)	≥37.5
**Sulter 2004**[1]	P	132	Ischaemic; Excl pts on antibiotics on admission	RC	<12 h; continuous; 48 h	Dichotomised according to hyperthermia or not within 48 h	baseline NIHSS, presence of infection, effect of antipyretics	>37.5
**Ernon 2006**[14]	R	107	Ischaemic, with thrombo-lysis	T/O	<180 mins; random; 24 h;	AUC relative to 37° and to baseline T°	NIHSS, 3 month mRS	>37.4
**Leira 2006**[11]	P	229	First ischaemic; Excl pts with inflammatory or infectious disease	A	<24 h; -; -	Presence vs. absence baseline pyrexia	CSS, BP, blood biochemistry, 4–7 d lesion volume (CT)	≥37.5
**Wong 2007**[15]	P	156	Ischaemic	T	<48 h (median 2.5 h); 4 h; 48 h	Mixed model, Lowess curves	Baseline NIHSS, use of paracetamol, presence of infection	None
**Idicula 2008**[16]	P	127	Ischaemic with thrombo-lysis	N/S	<3 h; random; 5 d	Pre-thombolysis temperature and peak temperature in 5 d	NIHSS, 3 month mRS, BP, peak [glucose]	>37.7
**Millan 2008**[17]	R	254	Ischaemic stroke with thrombolysis	N/S	<3 h; 6 h; 48 h	Pre-thrombolysis, temperature at 24 and 48 h, and peak temperature within 24 h post- thrombolysis	NIHSS, 3 month mRS, early lesion vol (CT), MCA TIBI score (TCD)	≥37
**Saini 2009**[7]	R	5305	First ischaemic	Most patients: A; others N/S	N/S; 8 then 24 hrly; 7 d	Normotherm vs. pyrexia at different time points	WCC, NIHSS, 3 month mRS, lesion vol (1–7 d CT/MR), use of antibiotics	>37.2
**den Hertog (PAIS) 2009 & 2011**[18,19]	P RCT	1399 or 1332	Any stroke <12 hrs	T or RC	median 6 h, all <12 h; -; -	Multiple logistic regression	baseline NIHSS, 14 d Barthel, 3 month mRS	None
**Naess 2010**[20]	R	250 (111 vs. 139)	Ischaemic	N/S	< 6 h; -; -	Logistic regression, temperature against outcome in tPA-treated vs. non-treated patients	baseline NIHSS, mRS on day 7 or at discharge, vascular risk factors, stroke aetiology	None
**Phipps 2011**[21]	R	1361	Ischaemic, NIHSS ≥ 2	Majority – T, partially unknown	<48 h; -; -	Logistic regression	vascular risk factors, NIHSS, stroke aetiology	≥37.8

P: prospective; SAH: subarachnoid haemorrhage; A: axillary; N/S: not specified; h: hours; d: days; CNS: Canadian Neurological Scale; GCS: Glasgow Coma Score; T: tympanic; SSS: Scandinavian Stroke Scale Score; WCC: white cell count; CSS: Canadian Stroke Scale; R: retrospective; RC: rectal; CT: computed tomography; mRS: modified Rankin Score; O: oral; NIHSS: National Institutes of Health Stroke Scale; CRP: C reactive protein; MR: magnetic resonance; AUC: area under the curve; BP: blood pressure; MCA: middle cerebral artery; TIBI: thrombolysis in brain ischaemia; TCD: transcranial Doppler; tPA: tissue plasminogen activator.

Amongst the 18 previous studies of temperature after stroke (Tables 1 and 2), four performed only one temperature recording [3,6,12,20], some measured temperature at admission but relatively late (e.g. 24 hours) [11] or at unspecified times after stroke [2,4,7]. Where serial readings were obtained, this was only up to three days after stroke [8,9,13,15,18,22]. Six studies included haemorrhagic and ischemic stroke [2-4,6,12,19]. Definitions of pyrexia, temperature measurement methods, reporting of associations with stroke severity and of causes of pyrexia varied widely [2,7,10,14,16,17,21]. Thus, while some studies found associations between higher admission temperature and poor outcome (Table 2) [3,6,12], others found that peak temperature occurring several days after stroke, not admission temperature, was associated with poor outcome [7,16,17,19]. The reasons for these differences are unclear but important to understand to limit brain damage and improve outcome after stroke.

**Table 2:** Previous studies of body temperature and outcome after stoke – key results

**First author and year**	**Key findings**
**Azzimondi 1995**[2]	High fever (≥37.9°C) <7 d is independent risk factor for poor prognosis. Fever occurred in 43% of stroke pts <7 d. Onset of fever occurred in first 2 days in 64% of febrile patients.
**Reith 1996**[3]	Admission body temp is independently related to stroke severity, lesion size, mortality and outcome. [unclear how measured “outcome”; didn’t separate AIS from ICH]
**Castillo 1998**[9]	The relationship between the degree of hyperthermia and stroke outcome/FIV is strongest when it begins within 24 h of symptom onset.
**Georgilis 1999**[4]	Fever in stroke is assoc with ↑age, ↑severity, more invasive techniques, worse outcome. When fever present without focus of infection, it tends to occur earlier.
**Wang 2000**[12]	For ischaemic stroke, admission temp (time unspecified) was significant predictor of in-hospital mortality: for each 1° increase, OR ↑ by 3.9 (CI 1.9 to 7.8, p<0.001).
**Boysen 2001**[8]	Temp < 6 h post stroke onset has no prognostic influence on 3 month mRS. More severe strokes have higher temperature in first 48 h. [Also looked at ICH]. 7 d fatality rate higher in patients with lower body temp on admission.
**Kammersgaard 2002**[6]	For all strokes, a 1° difference in admission body temperature gives 30% increase in relative risk of 5 yr mortality. No association between admission temp and survival in pts still alive at 3 months.
**Audebert 2004**[13]	Larger stroke volume and greater NIHSS assoc with higher temp, CRP and WCC. Successful thrombolysis attenuates inflammatory response
**Sulter 2004**[1]	56% developed hyperthermia in 1^st^ 48 h. Infectious cause found in 1/3 of patients.
**Ernon 2006**[14]	Hyperthermia relative to baseline in 24 h (post rtPA) is assoc with unfavourable outcome
**Leira 2006**[11]	Hyperthermia assoc with higher levels of proinflammatory markers. Inflammatory mediators play a role in acute ischaemic brain damage independently of hyperthermia
**Wong 2007**[15]	Mean temp rise in first 24 h from 36.5 to 36.7°, peak at 36 h. More severe strokes have higher temp rise.
**Idicula 2008**[16]	Body temp before thrombolysis was not assoc with 3 month outcome, but high temp thereafter was.
**Millan 2008**[17]	Body temp ≥37 at 24 h but not at baseline was assoc with lack of recanalisation, greater hyperdensity volume and worse functional outcome, regardless of stroke severity and time to treatment
**Saini 2009**[7]	Hyperthermia assoc with poor outcome. Delayed hyperthermia is more strongly assoc with poor outcomes than early hyperthermia. No association between baseline hyperthermia and outcome.
**den Hertog (PAIS) 2009 & 2011**[18,19]	Baseline body temp was not related to improvement. Increased body temp at 24 h was associated with low likelihood of improvement.
**Naess 2010**[20]	High body temperature was associated with favorable short-term outcome in those who were thrombolysed vs. those not thrombolysed
**Phipps 2011**[21]	High “fever burden” (combination of fever height and duration) was associated with death or with referral to hospice

d: days; AIS: acute ischemic stroke; ICH: intracerebral haemorrhage; FIV: final infarct volume; h: hours; OR: odds ratio; CI: confidence interval; mRS: modified Rankin Score; NIHSS: National Institutes of Health Stroke Scale; CRP: C reactive protein; WCC: white cell count; rtPA: recombinant tissue plasminogen activator.

We obtained measurements of body temperature every four hours from admission to five days after acute ischemic stroke as part of a study to examine serial changes in the ischaemic lesion on MR imaging. We used these data to clarify the temporal profile, associations with stroke severity, subtype and functional outcome and proportion with an alternative explanation for pyrexia.

## Methods

We prospectively recruited patients >18 years old who presented with potentially disabling ischemic stroke. We excluded patients with intracerebral haemorrhage (ICH), coma, serious intercurrent illness, and technical or clinical incompatibility with MR scanning. Ethical approval was granted by the Scottish Multi-Centre Research Ethics Committee (06/MRE00/119) and we obtained informed, written consent from all patients or their relatives.

At admission, we recorded National Institute of Health Stroke Score (NIHSS) and the Oxford Community Stroke Project (OCSP) stroke subtype classification [23]. All patients underwent MR imaging to diagnose the ischemic stroke. We recorded temperature using a First Temp Genius® tympanic thermometer immediately on arrival at hospital, and four hourly thereafter up to 120 hours. Recordings were taken from the uppermost ear in most patients, and from both ears in 11 patients to test side-to-side variation [24]. Tympanic thermometry is considered reliable for serial readings [25], shows the least variation with age compared with axillary, rectal and oral thermometry [26], and is widely used in clinical practice. Pyrexia was defined as temperature ≥37.5°C [10]. We searched for causes of pyrexia by collecting data on evidence of infection (evident infectious agent, leukocytes in body fluid, signs on imaging), DVT and surgical intervention as well as paracetamol and antibiotic use. Our policy is to prescribe paracetamol to patients who develop pyrexia (or for other indications such as pain relief) and antibiotics when positive evidence of infection and an infectious agent is diagnosed. We recorded modified Rankin Scale score (mRS) at three months after stroke, blind to temperature measurements.

We compared admission, peak and final temperatures and calculated area under the temperature/time curve (AUC)[27] which we standardised (AUC_[s]_) by assuming all patients had a temperature of 36.5°C between stroke and the admission recording, and dividing AUC by the time of final temperature reading [27]. We tested the association between temperature profile and measures of stroke severity firstly as determined by NIHSS, secondly by comparing total anterior circulation strokes (TACS) vs. less severe stroke subtypes (non-TACS) and thirdly good (mRS ≤2) vs. poor (mRS≥3) 90 day outcome. We adjusted analysis for age and admission NIHSS where appropriate. We used Student’s t-test for parametric and Mann Whitney U tests and Spearman correlation for non-parametric data.

## Results

Between 7^th^ December 2007 and 24^th^ March 2009, we recruited 48 patients but excluded two with ICH, one with complex migraine and one with functional limb weakness, leaving 44 with acute ischemic stroke (21 males) of median age 74 years (range 37–88) and median admission NIHSS 7 (range 0–28). There were 14 TACS and 30 non-TACS (Table 3). Twenty-five patients had poor (mRS≥3) and 19 had good (mRS≤2) 90 day functional outcomes. Patients with poor outcome were older (75±10) with higher admission NIHSS (13±7) than patients with good outcome (68±11 and 5±4 respectively).

**Table 3:** Baseline data, pyrogenic factors and use of antibiotics and thrombolysis

		**Total n=44**	**No pyrexia n=28**	**Pyrexia n=16**	
**Sex**					
	Male	21	15	6	
	Female	23	13	10	
**Mean Age in years, (SD)**		71.9 (11.4)	71.9 (11.7)	71.9 (11.3)	p=0.990
**Stroke Subtype**					
	TACS	14	5	9	TACS vs
	PACS	19	14	5	non-TACS
	LACS	6	6	0	χ^2^=6.9
	POCS	5	3	2	p=0.009
**Stroke severity**					
	Median NIHSS (IQR)	7 (3–14)	6.5 (3–10)	12 (5–18)	p=0.0381 (MWU)
**Temperature**					
	Time to peak, median hours after stroke, (IQR)	35.5 (19–53.8)	36.0 (7.8-59.3)	32.5 (22.8-51.3)	p=0.652 (MWU)
**N**^**o**^. with ≥1 pyrogenic factor identified*		16 (36%)	7 (25%)	9 (56%)	
	Urinary catheter	8	3	5	
	NG tube	9	4	5	
	Surgical procedure	4	2	2	
	DVT	0	0	0	
	Infection: urinary	2	0	2	
	Infection: respiratory	7	4	3	
**Antibiotics**		10	4 (14%)	6 (38%)	
**Paracetamol**		13	7 (25%)	6 (38%)	
**Thrombolysis**		3	1 (4%)	2 (13%)	
**90 day mRS**					
	mRS ≤2	19	15	4	χ^2^=3.5
	mRS ≥3	25	13	12	p=0.061

*Some patients had more than 1 potential cause of pyrexia. Pyrexia was defined as tympanic temperature ≥37.5°C. SD: standard deviation; TACS: total anterior circulation stroke; PACS: partial anterior circulation stroke; LACS: lacunar stroke; POCS: posterior circulation stroke; NIHSS: National Institute of Health Stroke Score; IQR: interquartile range; MWU: Mann Whitney U test; NG: nasogastric; DVT: deep venous thrombosis; mRS: modified Rankin Score.

There was no difference between ipsi- and contralateral tympanic temperature readings obtained simultaneously (36.6°C vs 36.6°C, 95% confidence interval (CI) -0.19 to 0.16, p=0.821) in 11 patients (five TACS). The mean number of temperature readings per patient was 14 (SD 6.9). No patients died during the observation period.

When temperature was first measured on admission, at a median 4 (interquartile range (IQR) 2 to 7.8) hours after stroke, all patients were normothermic (mean 36.4°C, 95% CI 36.2 to 36.6) (Table 3). Peak temperature (mean 37.3°C, 95% CI 37.1 to 37.5) occurred at median 35.5 hours (IQR 19.0-53.8) after stroke. The latest temperature (mean 36.5°C, 95% CI 36.3 to 36.7) was recorded at median 108.5 hours (IQR 98.8-113.5) after stroke.

Admission NIHSS score was not associated with admission temperature (adjusted r^2^=0.0, p=0.353), but was associated with peak (adjusted r^2^=0.20, p=0.002), and final (adjusted r^2^=0.25, p=0.001) temperatures. NIHSS was also associated with the overall temperature profile AUC[s] (adjusted r^2^=0.07, p=0.047).

**Figure 1:**
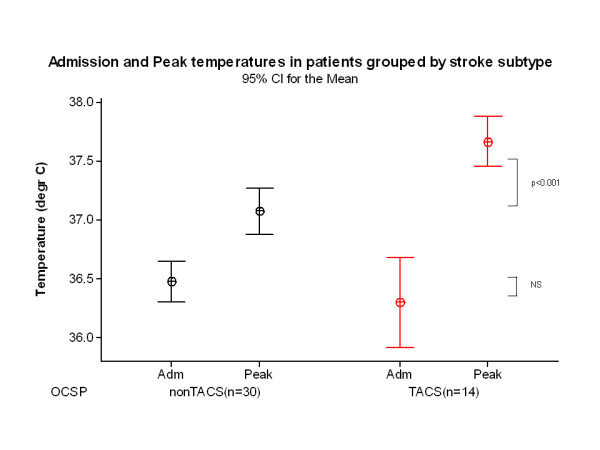
Admission, peak and final temperature in TACS (n=14) and non-TACS patients (n=30).

**Figure 2:**
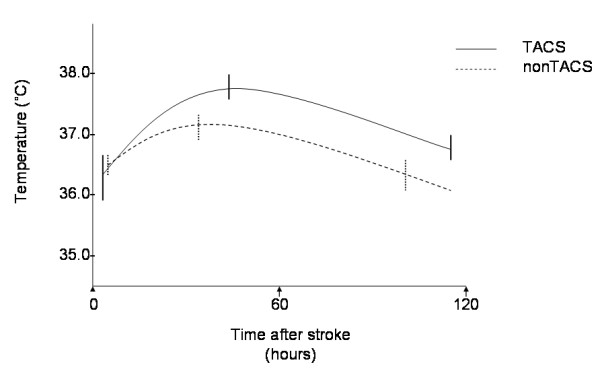
**Body temperature profiles of patients with severe stroke (TACS) and milder stroke (non-TACS).** Mean temperatures and 95% confidence intervals are shown for admission, peak and final temperature readings, averaged across all patients.

Admission temperature in TACS (mean 36.3°C, 95% CI 35.9-36.7) was similar to temperature in non-TACS (36.5°C, 95% CI 36.3-36.6) patients (p=0.382), measured at median 3.5 hours (IQR 1.0-6.0) in TACS, and 4 hours (IQR 2.0-8.8) in non-TACS (p=0.258, Mann Whitney U test), Table 4, Figure 1. However, peak temperature was higher in TACS (mean 37.7°C, 95% CI 37.5-37.9) than in non-TACS (37.1°C, 95% CI 36.9-37.3, p<0.001), occurring at median 46.0 hours in TACS and 32.5 hours in non-TACS (p=0.199, Mann Whitney U test). Final temperature was also higher in TACS (mean 36.8°C, 95% CI 36.6-37.0) than non-TACS (36.3°C, 95% CI 36.1-36.6, p=0.002) patients, and occurred later in TACS (median 117.5 hours, IQR 107.5-119.8) than in non-TACS patients (107.5 hours, IQR 94.3-111.5, p=0.007 Mann Whitney U test). Consequently, TACS patients had a larger AUC_[s]_ (36.6, 95% CI 36.5-36.8) than non-TACS patients (36.4, 95% CI 36.3-36.5, p=0.019). This is demonstrated graphically in Figure 2. Detailed temperature profiles for TACS and non-TACS patients are provided in Additional file 1: Figure S1a and b.

**Table 4:** Mean admission, peak and final body temperatures in patients grouped according to OCSP classification

**Stroke subtype (OCSP)**	**n**	**Admission temperature °C (95% CI)**	**Peak temperature °C (95% CI)**	**Final temperature °C (95% CI)**	**90 day mRS 0 to 2 (n)**	**90 day mRS 3 to 6 (n)**
TACS	14	36.3 (35.9-36.7)	37.7 (37.5-37.9)	36.8 (36.6-37.0)	2	12
PACS	19	36.4 (36.2-36.6)	37.0 (36.8-37.3)	36.3 (36.0-36.7)	11	8
LACS	6	36.7 (36.4-36.9)	37.0 (36.7-37.2)	36.2 (36.0-36.3)	3	3
POCS	5	36.6 (35.8-37.4)	37.3 (36.5-38.1)	36.5 (36.0-36.9)	3	2
**Total**	**44**	**36.4 (36.3-36.6)**	**37.3 (37.1-37.4)**	**36.5 (36.3-36.7)**	**19**	**25**

OCSP: Oxford Community Stroke Project; CI: confidence interval; TACS: total anterior circulation stroke; PACS: partial anterior circulation stroke; LACS: lacunar stroke; POCS: posterior circulation stroke; mRS: modified Rankin Score.

**Figure 3:**
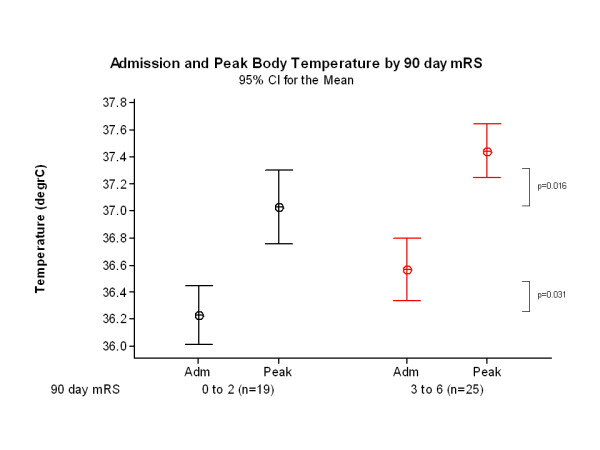
Admission, peak and final temperature by 90 day outcome (mRS ≤2, n=19; mRS ≥3, n=25).

Admission temperature was higher in the 25 patients with poor outcome (36.6°C, 95% CI 36.3-36.8) than in the 19 patients with good outcome (36.2°C, 95% CI 36.0-36.4, p=0.031) at 90 days. Peak temperature was also higher in patients with poor outcome (37.4°C, 95% CI 37.2-37.6) than in patients with good outcome, (37.0°C, 95% CI 36.8-37.3, p=0.016) (Figure 3). A higher proportion of patients with pyrexia (12/16, 75%) had a poor 90 day outcome than did patients without pyrexia (13/28, 46%), odds ratio (OR) 3.46, 95% CI 0.89 to 13.39) (Table 3).

Sixteen (36%) patients became pyrexial (at least one temperature reading of ≥37.5°C) during the recording period: 9/14 (64%) TACS patients and 7/30 (23%) non-TACS (χ^2^=6.9, p=0.009). Of the 16 pyrexial patients, at least one potential cause of pyrexia was identified in nine (56%), but 11/16 (69%) had no infection and 7/16 (44%) had no identified cause for pyrexia (apart from the stroke). Conversely, a potential pyrogenic factor was identified in 7/28 (25%) patients without pyrexia (Table 3). Thirteen patients were prescribed paracetamol of whom seven were recorded as having pyrexia and six were not (OR for pyrexia associated with paracetamol=1.8, 95% CI 0.48 to 6.77). The reason for paracetamol administration in the patients without pyrexia was mostly for pain relief. Ten patients with poor outcome (40%) had been prescribed paracetamol but only in three (16%) patients with good outcome (OR for poor outcome 3.56, 95% CI 0.82 to 15.46).

## Discussion

In patients with ischemic stroke, tympanic temperature was not elevated on admission even in patients with more severe strokes (TACS), and admission temperature did not correlate with admission NIHSS. Instead, we found that peak temperature, occurring at around 1.5 to 2 days after stroke and overall temperature, as expressed by AUC were associated with admission stroke severity as measured by NIHSS and TACS subtype. Peak temperatures were higher, occurred later and temperature elevation lasted longer in more severe than less severe strokes perhaps indicating a more prolonged, greater inflammatory response to the volume of infarcted tissue. Patients with poor functional outcome (mRS≥3) at 90 days had higher admission and peak temperatures than patients with good outcome (mRS≤2), although all admission temperatures were <37°C. We were not able to find a source of infection in 69% of pyrexial patients, and no alternative cause of pyrexia, other than the stroke itself, in 44%.

This prospective study of detailed four-hourly tympanic temperature measurements up to five days after ischemic stroke helps explain the varying results of previous studies (Tables 1 and 2) some of which found associations between admission temperature, stroke severity and outcome [3,4,6,12,13], while others did not [8,14,16,19]. This variation can be attributed to some studies being retrospective, or not specifying the timing of temperature recording after stroke, sampling temperature only once, measuring “admission” temperature relatively late after stroke, only measuring serial temperature up to 72 hours, the different severities of stroke included in each study or including patients with both haemorrhagic and ischemic stroke. Consistent with our results, others showed that patients with more severe strokes experience higher peak temperatures [15], and that elevated temperature at 24 hours [14,17,19], 48 hours [8] or 7 days [7] after stroke was more closely linked to poor outcome than admission readings. Our detailed longitudinal findings also demonstrate the higher, later and longer duration of temperature elevation in more severe than less severe stroke, which perhaps has not been appreciated previously.

Our study has limitations. The small sample size was constrained by selection of patients for an MR imaging study, but on the other hand it allowed very detailed temperature monitoring. However, the range of stroke severity was consistent with those that would be considered for trials of therapeutic hypothermia. A larger sample size would allow more adjustment for potential confounders, and clarification of the significance of any differences in timing of peak temperature readings, and comparison of patients whose tympanic temperature may have been affected by antibiotics and antipyretics, although our results show that pyrexia was just as common in patients who were prescribed paracetamol as in those who were not, in contrast to others’ results [15]. However paracetamol may have influenced the profile of temperature change. Although our data on temperature profile and stroke severity are consistent with five previous studies, we cannot exclude the possibility of an association between admission temperature and stroke severity. The study strengths are the detailed four-hourly tympanic temperature measurements for 120 hours after ischemic stroke and the detailed comparison with stroke subtype, severity and outcome. The duration of temperature recording ensured that the peak temperature was captured in both TACS and non-TACS.

Two other points raise questions for further study. Admission temperature in the patients with more severe strokes, ie TACS, was not higher than in patients with milder strokes, as might have been expected. Perhaps, by analogy with other serious acute illness, the temperature in severe stroke may reflect severe illness [28]. Secondly, why do patients with a poor outcome have a marginally higher (but still normothermic) admission temperature, and while severe stroke is associated with poor outcome, severe stroke is not associated with admission temperature? This might be explained at least in part by a possible cascade of events suggested in experimental data. Increased temperature opens the blood–brain barrier [29] which, in acute ischemia, would lead to increased extracellular oedema, more infarct swelling, more restricted capillary flow in the ischemic tissue, less chance of reperfusion, all contributing to increasing ischemic damage, swelling and leading to a larger infarct around 48 hours [30], consolidating the potential for tissue damage that was suggested by the severe stroke symptoms at presentation, and consequently leading to the poor outcome at 90 days. Longer duration of temperature monitoring should be considered in future research to improve understanding of temperature profiles after stroke.

This interpretation, if true, raises some implications for therapeutic hypothermia trials. Firstly, and paradoxically, patients may benefit the most from hypothermia if it prevents the tissue cascade outlined above from causing more tissue damage and should certainly not be excluded from trials; thus cooling should be initiated as early as possible and not influenced by the patient’s admission temperature. However cooling may only prevent worsening of damage, not actively salvage tissue that is already at risk, so combinations of therapies to salvage (e.g. thrombolysis) as well as to restrict progressive new damage (e.g. hypothermia, if it works) may be required. Secondly, if the main effect of hypothermia is to reduce blood–brain barrier opening thus preventing the cascade that leads to larger stroke lesions, then hypothermia could still be valuable if started many hours after the stroke when the possibility of salvaging at risk tissue was lost but there was still some “future secondary damage” to prevent. If correct, then hypothermia should reduce subacute infarct oedema and mass effect. Thirdly, if correct, then therapeutic thrombolysis and hypothermia should work synergistically to produce greater benefit than either alone. However hypothermia may also have some disadvantages. In addition to increased risk of secondary infection and the need to manage unpleasant side effects like shivering, lower temperatures might delay thrombus lysis which pyrexia might accelerate. The balance of risk and benefit presented by these possibilities requires testing in future therapeutic trials of hypothermia after stroke.

## Conclusion

The association between body temperature, stroke severity and functional outcome is complex and, if our observations in this small detailed study are verified, then even very marginal differences in admission temperature in normothermic patients may provide a mechanism whereby, for a given stroke severity, potential tissue damage is converted into more severe damage resulting in a poor outcome, which might be prevented by therapeutic hypothermia.

## Competing interests

J Wardlaw is the Imaging Workpackage Lead for the EuroHyp trial of therapeutic hypothermia in stroke. The work was conducted prior to and independently of the EuroHyp trial. The authors have no other competing interests.

## Authors’ contributions

RGR Thomas and B Karaszewski recruited patients, collected and entered data in to the database and performed initial analysis. MS Dennis supervised the clinical stroke examination and subtyping. JM Wardlaw initiated and supervised the project, checked the data, and with RG Thomas, drafted and with all authors, edited the manuscript. All authors have read and approved the final manuscript.

## Pre-publication history

The pre-publication history for this paper can be accessed here:

http://www.biomedcentral.com/1471-2377/12/123/prepub

## Supplementary Material

Additional file 1 **Figure S1.** (online only). Individual patient body temperature profiles for (a) non-TACS and (b) non-TACS patients. These graphs show mean body temperature values over 120 hours after stroke onset with nonlinear regression curves. Click here for file
