# Supplementary figures and images for: Temporal profile of body temperature in acute ischemic stroke: relation to stroke severity and outcome
Source: BMC Neurol. 2012 Oct 18;12:123. doi: 10.1186/1471-2377-12-123 (PMC3607983; doi:10.1186/1471-2377-12-123)

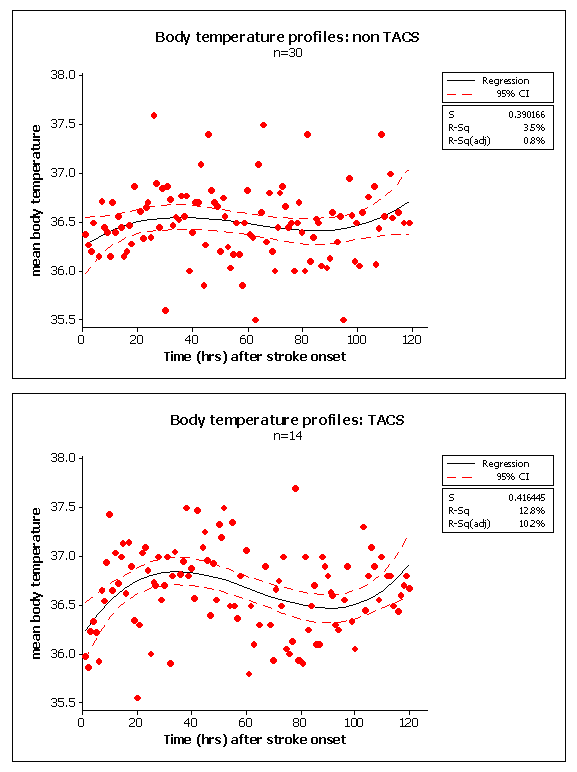

Supplement: Additional file 1 — Figure S1. (online only). Individual patient body temperature profiles for (a) non-TACS and (b) non-TACS patients. These graphs show mean body temperature values over 120 hours after stroke onset with nonlinear regression curves. [file 1471-2377-12-123-S1.tiff]
